# Lipid nanoparticle technology-mediated therapeutic gene manipulation in the eyes

**DOI:** 10.1016/j.omtn.2024.102236

**Published:** 2024-06-03

**Authors:** Ting Wang, Tao Yu, Qian Liu, Tzu-Cheng Sung, Akon Higuchi

**Affiliations:** 1 State Key Laboratory of Ophthalmology, Optometry and Visual Science, Eye Hospital, Wenzhou Medical University, No. 270, Xueyuan Road, Wenzhou, Zhejiang 325027, China; 2 Department of Chemical and Materials Engineering, National Central University, No. 300, Jhongda RD, Jhongli, Taoyuan 32001, Taiwan

**Keywords:** MT: Delivery Strategies, nucleic acid-based drugs, ocular diseases, lipid nanoparticles, LNP technology, ocular gene therapy

## Abstract

Millions of people worldwide have hereditary genetic disorders, trauma, infectious diseases, or cancer of the eyes, and many of these eye diseases lead to irreversible blindness, which is a major public health burden. The eye is a relatively small and immune-privileged organ. The use of nucleic acid-based drugs to manipulate malfunctioning genes that target the root of ocular diseases is regarded as a therapeutic approach with great promise. However, there are still some challenges for utilizing nucleic acid therapeutics *in vivo* because of certain unfavorable characteristics, such as instability, biological carrier-dependent cellular uptake, short pharmacokinetic profiles *in vivo* (RNA), and on-target and off-target side effects (DNA). The development of lipid nanoparticles (LNPs) as gene vehicles is revolutionary progress that has contributed the clinical application of nucleic acid therapeutics. LNPs have the capability to entrap and transport various genetic materials such as small interfering RNA, mRNA, DNA, and gene editing complexes. This opens up avenues for addressing ocular diseases through the suppression of pathogenic genes, the expression of therapeutic proteins, or the correction of genetic defects. Here, we delve into the cutting-edge LNP technology for ocular gene therapy, encompassing formulation designs, preclinical development, and clinical translation.

## Graphical abstract

**Figure:**
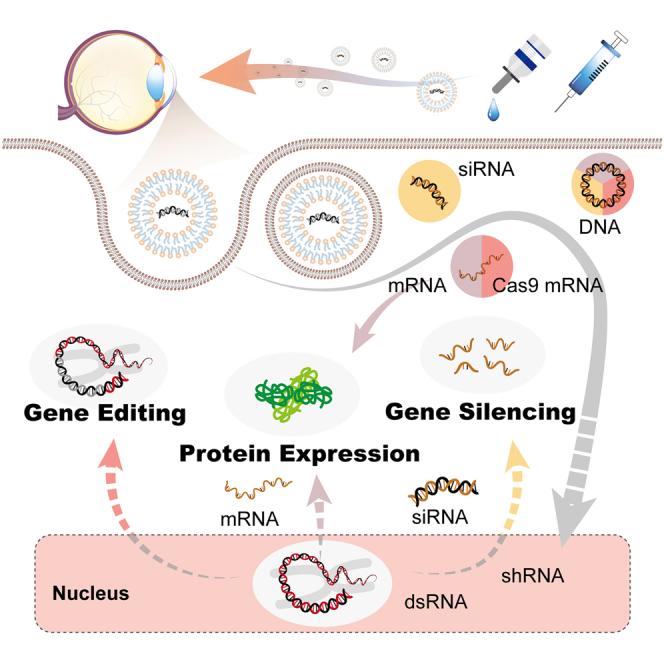

## Teaser

Higuchi and colleagues review advanced LNP technology for ocular gene therapy, covering formulation design, preclinical development, and clinical translation. They emphasize the key questions that remain in the realm of ocular gene therapy and provide a perspective on the future of LNP-based nucleic acid therapies for ocular diseases.

## Introduction

**Figure 1:**
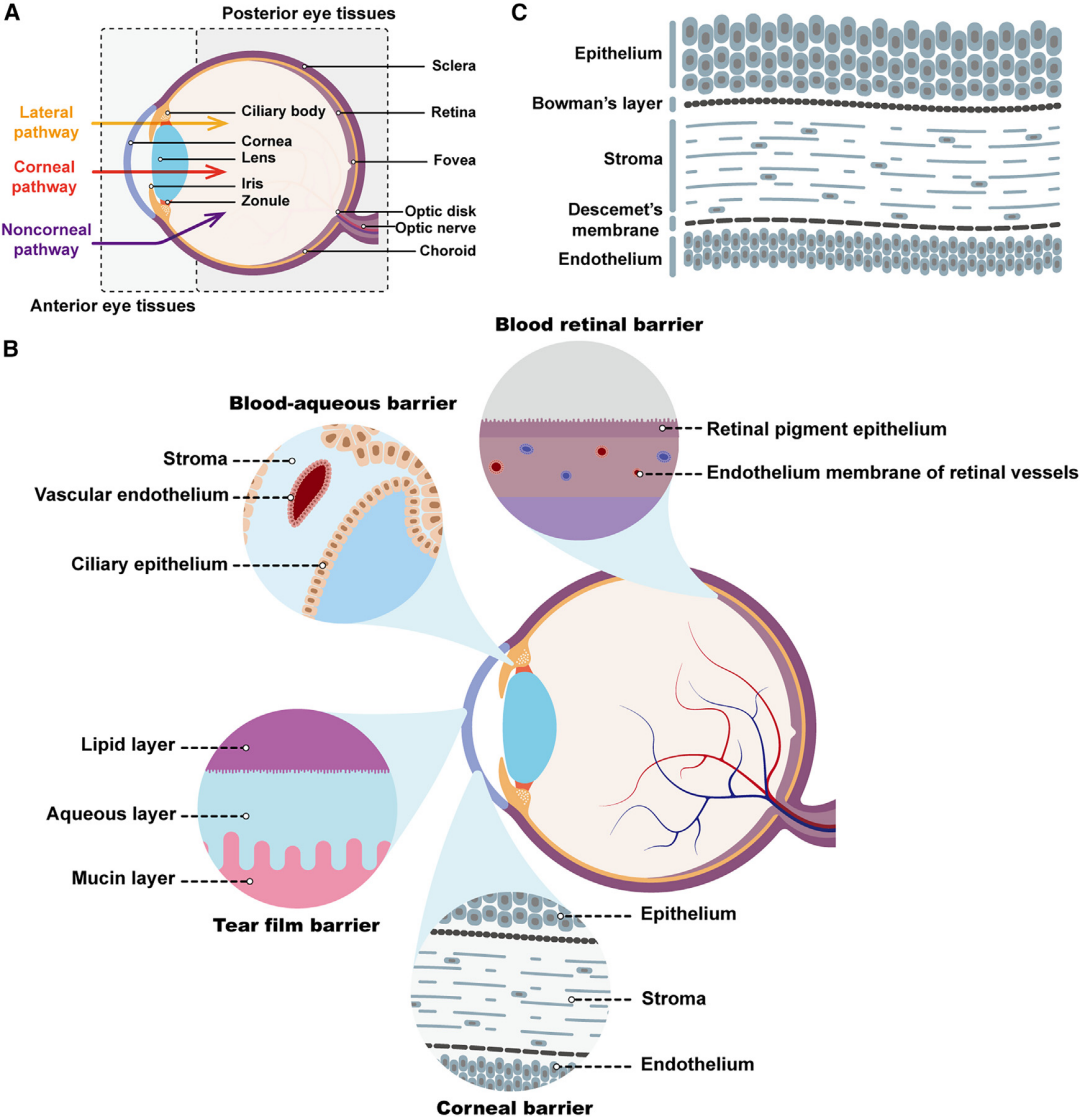
Eye structure Diagram of anatomical components (A), physiological barriers (B), and corneal layers (C) in ocular tissues. (B) Schematic representation of four physiological barriers, which include the tear film barrier, corneal barrier, blood-aqueous barrier (BAB), and blood-retinal barrier (BRB). (C) Schematic representation of the corneal layers with additional Bowman’s layer and Descemet’s membrane detailed.

The eye is one of the most important and unique sensory organs of the human body and is regarded as the window to the brain or window to one’s soul.^1^ The anatomical structure of the eyes is complex and delicate, and disruption of any part of the ocular tissues could cause ocular discomfort and lead to damage of visual performance or even loss of vision (Figure 1A). Many people suffer from eye disease and/or vision loss. Certain inherited ocular diseases, such as Leber hereditary optic neuropathy, Leber congenital amaurosis (LCA), and X-linked retinoschisis (XLRS), result from genetic disorders of the eyes. Furthermore, they have a higher risk of becoming common age-related eye diseases, including age-related macular degeneration (AMD), cataracts, and glaucoma, as people age.^2^ The prevalence of these types of diseases is continuously increasing as the aged population continues to increase worldwide. In addition, some genetic eye diseases also lead to complications of other eye diseases, such as diabetic retinopathy and thyroid-associated ophthalmopathy. These complex ocular diseases lead to faster vision impairment and blindness worldwide and require urgent treatment.

With the development of nucleic acid-based therapeutics as well an increasing understanding of the etiology and pathogenesis of certain kinds of eye diseases,^3^ the ability to treat ocular diseases by targeting specific disease-related genes (i.e., *RPE65*) is gradually becoming a reality in clinical use.^4^ Nucleic acid-based therapeutics can manipulate specific ocular diseases at the gene (DNA or RNA) level, which is quite different from small molecule-based therapeutics or biological agent-mediated therapeutics targeting gene products (i.e., proteins). Three types of strategies are primarily used in treating inherited or acquired ocular genetic disorders due to the versatility of nucleic acid-based therapeutics: (1) therapeutic DNA engineering-based gene editing strategies (correcting dysfunctional/mutated genes), (2) mRNA cargo-based therapeutics enhancing the duration and amplitude of disease-related therapeutic protein production, and (3) small interfering RNA (siRNA) therapy-induced effective gene silencing, which could cause the inhibition of pathological/mutant protein production. The first ocular gene replacement therapeutic, voretigene neparvovec (Luxturna), was approved by the U.S. Food and Drug Administration (FDA) in 2017, which represented a milestone for the treatment of inherited retinal degeneration and opened the golden era for ocular gene therapy.^4^ Voretigene neparvovec included an adeno-associated virus 2 (AAV2) vector containing human *RPE65* cDNA with a modified Kozak sequence that treats *RPE65*-associated LCA.^5^

Except for voretigene neparvovec, various nucleic acid-based therapeutics are currently undergoing diverse stages of clinical evaluation. Owing to the nature and unfavorable properties of nucleic acids, the therapeutic application of nucleic acids *in vivo* is challenging.^6^ Specifically, the negatively charged nature of nucleic acids makes it challenging for them to traverse negatively charged cell membranes, and their susceptibility to degradation by nucleases in the circulation further complicates matters. In addition to elevated chemical modification techniques to improve the physicochemical characteristics and the stability of nucleic acids, advanced ideal delivery technologies (nanocarriers or vectors) are necessary to promote targeted tissue accumulation, cellular internalization, and enhancement of the targeting affinity of nucleic acids.

In voretigene neparvovec, therapeutic human *RPE65* cDNA is delivered to the subretinal space using AAV vectors following a subretinal injection.^4^ However, fast and broad implementation of AAV-based gene therapy for further ocular genetic therapeutics has been hampered by several factors. Firstly, conventional AAV vectors have a limited packaging capacity of approximately 5 kb, which restricts the size of the genetic payload that can be delivered. Additionally, the production costs associated with AAV-based gene therapy are high, which stem from various factors, including extensive research and development, intricate manufacturing processes, and rigorous quality control measures.^7^ Limited scale of production also contributes to high per-unit costs.^7^ For instance, voretigene neparvovec costs $850,000 per one-time treatment, excluding expenses such as surgery (estimated at $4,876) and other medical costs.^8^ Indeed, the high price of voretigene neparvovec poses a significant burden, not only for patients without coverage by healthcare systems, but also for insurance systems that cover the costs. Moreover, there is a potential risk of integration into the host genome, known as insertional mutagenesis, which has raised safety concerns. It is important to note that, to date, there have been no confirmed reports of genotoxic events resulting from recombinant AAV-mediated insertional mutagenesis in humans.^9^ However, it is essential to acknowledge this theoretical risk, especially considering that insertional mutagenesis has been observed in a small number of murine studies.^9^^,^^10^^,^^11^^,^^12^

Lipid nanoparticle (LNP) platforms stand as one of the most advanced nonviral synthetic vehicles, playing a pivotal role in enabling the application of nucleic acid-based therapeutics.^10^^,^^11^^,^^12^^,^^13^ Compared with virus-type vectors, LNPs are easier to produce and modify by verifying the molecular structure, which decreases production costs. LNPs can load and deliver up to 20 kb of DNA and mRNA (i.e., *ABCA4* or *USH2A*),^14^ which are too large to be accommodated in virus-type vectors.^15^^,^^16^ In addition, unlike viral vectors, therapeutic RNA encapsulated in LNPs avoids integration into the host cell’s genome, thereby mitigating the risk of insertional mutagenesis. The advancements in lipid-based delivery systems that were originally intended for small molecular therapeutics in recent decades has made great contributions to the application of LNP technology for nucleic acid delivery in recent years. These efforts included systematically optimizing LNP components for efficient gene silencing and incorporating siRNA payload modification and chemistry using polyethylene glycol (PEG) lipids, helper lipids, and, particularly, cationic or ionizable lipids. The bravura mix of priming, advanced LNPs and burgeoning RNA interference (RNAi) contributed to the creation and subsequent FDA approval of patisiran (Onpattro).^17^ Patisiran holds the distinction of being the first LNP-delivered RNAi drug officially approved by the FDA and is used to treat hereditary amyloidogenic transthyretin (TTR) amyloidosis. The LNP platform is used in patisiran for efficient delivery of TTR siRNA to hepatocytes after systemic infusion, which inhibits the mutant TTR protein production and prevents the subsequent fibrils formation.

This review offers a comprehensive overview of lipid nanotechnology-mediated gene manipulation approaches employed in both clinical and preclinical research for the treatment of diverse ocular diseases. First, we describe the eye microanatomy and barriers, which indicate the advantages of topical gene therapy in ocular applications. Second, the reported major mutation genes related to different major ocular diseases (inherent retinal diseases, age-related ocular diseases) are summarized. In addition, several clinical trials for patients with different kinds of ocular diseases are summarized. The identification of genetic factors in the majority of eye diseases provides an array of potential targets for gene replacement, gene knockdown, or gene editing therapies, which could be delivered by ideal delivery vectors. Third, we delve into the design criteria and production methods, focusing on specific research related to the administration of LNP-mediated nucleic acid therapeutics to diseased eyes. Finally, we underscore the significance of (pre)clinical development of LNP-mediated therapeutic nucleic acid drugs in the treatment of genetic ocular diseases and infections.

## Eye microanatomy, drug flow pathways, and barriers

The eye is a highly complex and unique organ, and it is anatomically divided into two parts by the lens plane: the anterior segment and the posterior segment (Figure 1A). Generally, drugs easily reach the anterior segment after non-invasive topical administration, such as eye drops or ointments, but have difficulty reaching the posterior segment.^18^ Recent reports indicate a significant increase in the prevalence of genetic disorders affecting the posterior eye segment.^19^^,^^20^ Current treatments for diseases occurring in the posterior eye segments mainly rely on surgery and different invasive techniques,^21^ which can cause a series of complications or adverse reactions (i.e., inflammation, subconjunctival hemorrhage) and cause poor compliance in patients. Comparatively speaking, topical administration of therapeutic drugs is a convenient and noninvasive way to treat ocular diseases in posterior eye segments.

Three primary routes are generally considered for achieving noninvasive local drug delivery to the posterior segment of the eyes after topical administration (Figure 1A).^22^^,^^23^^,^^24^^,^^25^^,^^26^ These routes include the corneal pathway, the conjunctival/scleral route (noncorneal pathway), and the lateral diffusion pathway. The corneal pathway is considered the primary route for drug delivery to the posterior segment of the eye after topical administration, where drugs flow through the cornea, aqueous humor, and vitreous before penetrating into the retina.^27^ The noncorneal pathway encompasses the diffusion of drugs through the conjunctiva, sclera, and choroid to reach the retina.^28^ The lateral diffusion pathway involves drugs diffusing from the cornea to the uveal and scleral tissues through the aqueous humor. These traditional ophthalmic drug diffusion pathways face challenges in achieving sufficient bioavailability in the posterior segment, which are mainly caused by physiological barriers to drug penetration and delivery in the eyes (Figure 1B).^28^

Four physiological barriers exist in noninvasive topical administration. These barriers make the eyes a relatively immune-privileged site, which positively suppresses certain immune and inflammatory responses, but also lead to challenges for drug penetration.^29^^,^^30^ In the anterior segment, the first obstacle encountered by topically applied molecules is the tear film, which is the main barrier for attaining a therapeutic concentration after local administration.^31^ The secretion and drainage of tears in the tear film facilitate the easy removal of drugs from the ocular surface.^32^ Drugs that enter the cornea after crossing the tear membrane will meet the corneal barrier.

The cornea is composed of five layers of tissue, as shown in Figure 1C, and it is considered the first physical barrier for drug absorption in the corneal pathway (Figure 1A).^33^^,^^34^ The epithelium and endothelium of the cornea are two types of tightly connected cells that restrict the diffusion of macromolecules and water-soluble drugs through the cornea.^18^ The stroma is a barrier to lipophilic drugs^35^ because of the extracellular matrix. In addition, the continuous secretion and excretion of the aqueous humor make the drugs easily to be removed.^36^ Furthermore, ciliary epithelium, iridial epithelium, and endothelium of iridial blood vessels form the blood-aqueous barrier, which blocks drug delivery.^37^ The retinal pigment epithelium (RPE) and the endothelium of retinal blood vessels together constitute the blood-retinal barrier,^38^ creating a substantial obstacle for retinal drug delivery.^39^^,^^40^^,^^41^^,^^42^

Despite these barriers, the eye is still an accessible organ for the use of nucleic acid-based therapeutics. The relatively small size and compartmentalized structure of the eyes make it difficult to investigate disease pathogenesis and challenge ocular drug discovery, but also create opportunities for local drug delivery and noninvasive clinical treatment of ocular diseases.^43^ In contrast, the broad identification of genetic factors in most eye diseases, such as inherited retinal degeneration and AMD, offers a range of potential targets for gene replacement, knockdown, or editing therapies.^43^ Numerous relatively straightforward (pre)clinical trials focusing on nucleic acid-based therapeutics targeting specific ocular diseases have been implemented for the evaluation of drug effectiveness to recover visual function.

## Common eye disease-related pathogenic genes and clinical trials

**Figure 2:**
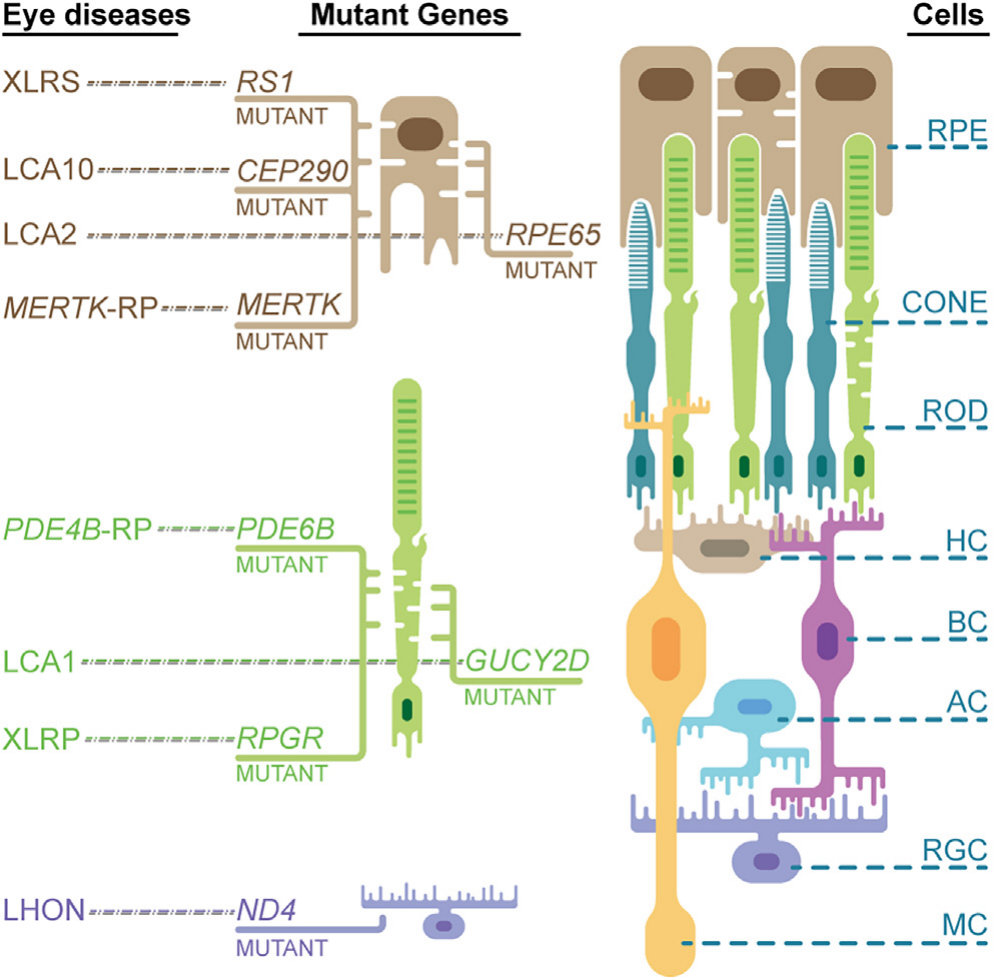
Schematic of representative inherited retinal degeneration or dystrophy and the related gene mutations The schematic on the left illustrates the major IRDs, which include X-linked juvenile retinoschisis (XLRS), LCA10, LCA2, *PDE6B*-associated retinal pigmentosa (*PDE6B*-RP), *MERTK*-associated retinal pigmentosa (*MERTK*-RP), LCA1, X-linked retinal pigmentosa (XLRP), and Leber hereditary optic neuropathy (LHON). The schematic on the right illustrates the major cell types of the retina and the RPE, including rod and cone PRs, bipolar cells (BCs), horizontal cells (HCs), amacrine cells (ACs), RGCs, and Müller glial cells (MCs). The cell types in the schematic are aligned with the corresponding affected cells in the immunohistochemically labeled retina.

A global estimate suggests that approximately 2.2 billion people suffer from vision impairment or blindness.^33^^,^^34^ Among these, one billion people have ocular defects that could have been prevented. The ocular defects are mainly caused by inherited retinal diseases (IRDs), choroideremia, Stargardt disease, XLRS, cataracts, AMD, glaucoma, and diabetic retinopathy.^44^^,^^45^^,^^46^^,^^47^^,^^48^^,^^49^ New techniques in next-generation sequencing have contributed to the comprehensive analysis of and significant discoveries in the etiology and pathogenesis of different types of ocular diseases in the last few decades,^50^^,^^51^^,^^52^ which are expected to pave the way for further ocular nucleic acid-based therapeutics. In this section, we will summarize several types of eye diseases (Figure 2) and their related mutated genes, as well as related clinical trials (Table 1).

**Table 1:** Clinical trials for ocular gene therapy

Interventions	Types	Diseases	Targets	Vector	Phase	Sponsor	ClinicalTrials.gov ID
Voretigene neparvovec	cDNA	RPE65 mutation-associated RP	*RPE65*	AAV2	FDA approved	Spark Therapeutics	NCT00516477
AAV2-hRPE65v2, voretigene neparvovec-rzyl	cDNA	LCA2	*RPE65*	AAV2	phase 3	Spark Therapeutics	NCT00999609
AAV2-hRPE65v2 Vector	cDNA	LCA2	*RPE65*	AAV2	phase 1/phase 2	Spark Therapeutics	NCT01208389
Safety Study of voretigene neparvovec-rzyl	cDNA	LCA2	*RPE65*	AAV2	phase 1	Spark Therapeutics	NCT00516477
rAAV2-CB-hRPE65	cDNA	LCA2	*RPE65*	AAV2	phase 1/phase 2	Applied Genetic Technologies Corp	NCT00749957
rAAV2/4.hRPE65	cDNA	LCA2	*RPE65*	AAV2	phase 1/ phase 2 completed	Nantes University Hospital	NCT01496040
AAV OPTIRPE65	cDNA	LCA2	*RPE65*	AAV2/5	phase 1/2 completed	MeiraGTx UK II Ltd	NCT02946879
AAV RPE65	cDNA	LCA2	*RPE65*	AAV2/5	phase 1/2 completed	MeiraGTx UK II Ltd	NCT02781480
rAAV2-hRPE65	cDNA	LCA2	*RPE65*	rAAV2	phase 1 completed	Hadassah Medical Organization	NCT00821340
rAAV2-CBSB-hRPE65	cDNA	LCA2	*RPE65*	rAAV2	phase 1	University of Pennsylvania	NCT00481546
EDIT-101	CRISPR	LCA10	*CEP290*	–	phase 1/phase 2	Editas Medicine, Inc.	NCT03872479
BIIB112 (XIRIUS)	cDNA	XLRP	*RPGR*	AAV8	phase 1/phase 2	NightstaRx Ltd, a Biogen Company	NCT03116113
rAAV2tYF-GRK1-RPGR	cDNA	XLRP	*RPGR*	AAV2	phase 1/phase 2	Applied Genetic Technolo gies Corp	NCT03316560
AAV2/5-RPGR	cDNA	XLRP	*RPGR*	AAV2/5	phase 1/phase 2	MeiraGTx	NCT03252847
AAV2/5-hPDE6B	cDNA	XLRP	*PDE6B*	AAV2/5	phase 1/phase 2	eyeDNA Therapeutics	NCT03328130
BIIB111	cDNA	Choroideremia	*CHM*	AAV2	phase 3	Biogen	NCT03496012
BIIB111 BIIB112	cDNA	Choroideremia	*CHM*	AAV2	phase 2	Biogen	NCT03507686
AAV2-hCHM	cDNA	choroideremia	*CHM*	AAV2	phase 1/phase 2	Spark Therapeutics	NCT02341807
4D-110	cDNA	choroideremia	*CHM*	AAV capsid variant	phase 1	4D Molecular Therapeutics	NCT04483440
tgAAG76	cDNA	retinal degeneration	*RPE65*	AAV2	phase 1/phase 2 completed	University College, London	NCT00643747
AAV2/5-hPDE6B	cDNA	RP-PDE6β	*5-hPDE6B*	AAV2	phase 1/phase 2	eyeDNA Therapeutics	NCT03328130
CPK850	cDNA	RLBP1 RP	*RLBP1*	AAV8	phase 1/phase 2	Novartis Pharmaceuticals	NCT03374657
rAAV2-VMD2-hMERTK	cDNA	MERTK-related RP	*MERTK*	rAAV2	phase 1 completed	King Khaled Eye Specialist Hospital	NCT01482195
AGTC-501	cDNA	X-linked RP	*RPGR*	rAAV2	phase 2/phase 3	Beacon Therapeutics	NCT04850118
4D-125	cDNA	X-linked RP	*RPGR*	4D-R100	phase 1/phase 2	4D Molecular Therapeutics	NCT04517149
RGX-314	cDNA	nAMD	anti-VEGF monoclonal antibody	AAV8	phase 2	AbbVie	NCT03999801
HMR59	cDNA	nAMD	sCD59	AAV2	phase 1	Janssen Research & Development, LLC	NCT03585556
ADVM-022	cDNA	nAMD	aflibercept	AAV2.7m8	phase 1	Adverum Biotechnologies	NCT03748784
QPI-100	siRNA	glaucoma	caspase-2 synthesized	–	phase 1 phase 2 phase 3	Quark Pharmaceuticals	NCT01965106 NCT01064505 NCT02341560
SYL040012	siRNA	glaucoma	β2 adrenergic receptors	–	phase 1/phase 2	Sylentis, S.A.	NCT02250612 NCT01739244 NCT00990743 NCT01227291
PF-04523655	siRNA	DME	*RTP801*	–	phase 2	Quark	NCT01445899
SYL1001 (Tivanisiran)	Naked siRNA	ocular pain	*TRPV1*	–	phase 3 completed	Sylentis	NCT05310422
Sepofarsen (QR-110)	mRNA ASO	LCA10	*CEP290* p.Cys998X	–	phase 2/phase 3	ProQR Therapeutics	NCT03913143
Ultevursen	ssRNA-based oligonucleotide	Usher syndrome type 2 related RP	*USH2A*	–	phase 2/phase 3	ProQR Therapeutics	NCT05158296
QR-1123	ASO	autosomal-dominant RP	mutant P23H mRNA	–	phase 1/phase 2	ProQR Therapeutics	NCT04123626
OPGx-001	undisclosed	LCA5-IRD	*hLCA5*	AAV8	phase 1/phase 2 not yet recruiting	Opus Genetics, Inc	NCT05616793
rAAV.hPDE6A	undisclosed	PDE6A-linked RP	*PDE6A*	rAAV	phase 1/phase 2	STZ eyetrial	NCT04611503
RST-001 optogenetics	undisclosed	advanced RP	–	–	phase 1/phase 2	AbbVie	NCT02556736
vMCO-010 Optogenetic	undisclosed	advanced RP	–	–	phase 2	Nanoscope Therapeutics Inc.	NCT04945772

rAAV, recombinant AAV.

### IRDs

Inherited retinal degeneration or dystrophy represents a diverse range of ocular diseases and serves as the predominant cause of currently untreatable blindness. This condition is characterized by the gradual deterioration of photoreceptors (PRs) and the RPE.^50^^,^^51^^,^^52^ Genetic factors play a significant pathogenic role in the development of retinal degeneration (Figure 2).

LCA is the most severe subtype of IRD, with a prevalence of approximately 1:50,000 in European and North American populations.^53^^,^^54^ LCA shows high genetic heterogeneity, and to date, mutations in 25 different genes have been associated with LCA (https://web.sph.uth.edu/RetNet/sum-dis.htm). Currently, there are more than 11 clinical trials (NCT00999609, NCT01208389, NCT00516477, NCT00749957, NCT00643747, NCT01496040, NCT02946879, NCT02781480, NCT00821340, and NCT00481546) related to LCA; detailed information is listed in Table 1.

### Stargardt disease

Stargardt disease is the most common form of juvenile macular degeneration and is the most prevalent form (approximately 90% of cases), which is predominantly inherited in a recessive manner and linked to mutations of one member of the ATP-binding cassette (ABC) transporter superfamily, the *ABCA4* gene in PR cells.^48^ Sanofi initiated a phase 1/2a half-dose escalation study of SAR422459 (NCT01367444) utilizing a lentivirus vector carrying the human *ABCA4* gene, which was prematurely terminated. A phase 1/2 follow-up study of SAR422459 (NCT01736592) and other neuroprotective clinical trials (NCT05417126) in patients with Stargardt’s macular degeneration is ongoing.

### Color vision deficiency

Color vision deficiencies are a group of vision disorders characterized by abnormal color discrimination.^55^ The color vision deficiencies include red-green color blindness, yellow-blue color blindness, and achromatopsia. The deficiencies are caused by mutations in the genes (*OPN1LW*, *OPN1MW*, *ATF6*, *CNGA3*, *CNGB3*, *GNAT2*, *PDE6H*, and *PDE6C*) coding for various components of retinal cones.^56^ Currently, phase 1/2 half-dose escalation trials (Table 1) targeting human *CNGB3* (NCT02599922 and NCT03278873) or *CNGA3* (NCT02935517 and NCT03758404) are ongoing and are sponsored by MeiraGTx.

### AMD

AMD is a leading cause of blindness in the developed world, especially in aging populations.^55^ AMD is, therefore, an important target for new therapeutic development. AMD affects an estimated 14 million people worldwide and is the leading cause of severe and irreversible vision loss in individuals older than 50 years in Western societies.^19^ AMD is an ideal target for gene therapy because of its high prevalence and because several genes are involved.^57^ Detailed information on some of the latest clinical trials on AMD (NCT03999801, NCT03585556, NCT03748784, and NCT01445899) is listed in Table 1.

### Glaucoma

Glaucoma comprises a group of optic neuropathies primarily distinguished by the progressive degeneration of retinal ganglion cells (RGCs) and their axons.^58^ Linkage analysis of heritable forms of glaucoma has identified 17 glaucoma loci, with six distinct loci (*GLC1A* through *GLC1F*) specifically associated with primary open-angle glaucoma.^58^ Detailed information on some of the latest clinical trials on glaucoma (NCT01965106, NCT01064505, NCT02341560, NCT02250612, NCT01739244, NCT00990743, and NCT01227291) is listed in Table 1.

## Preclinical development of lipid nanotechnology-mediated gene therapy for ocular disease

Gene therapy, encompassing gene insertion, gene editing, and gene silencing, emerges as a highly promising therapeutic approach for patients with ocular diseases. Its attractiveness lies in the potential to address and correct the underlying genomic malfunctions responsible for these conditions. The eye is an attractive target for *in vivo* gene therapy owing to its special characteristic features. Its relatively small size and physical barriers allow for topical administration of LNPs, and the immune-privileged property of the eyes is beneficial for decreasing inflammation when foreign biomaterials or cells are transplanted into the eyes. Furthermore, numerous retinal degenerative diseases are thoroughly characterized from a genetic perspective.^59^ Ocular gene therapy is still in its early stages, and related research is evolving rapidly. Advances in ocular genetic therapy have played a pivotal role in establishing new standards of care for patients with various eye diseases. Voretigene neparvovec-rzyl (Spark Therapeutics) is the first U.S. FDA-approved ocular gene therapy product for patients with biallelic *RPE65*-associated LCA or retinitis pigmentosa (RP).^60^ Voretigene neparvovec-rzyl has exhibited the safety and effectiveness of gene augmentation therapy, which utilizes an AAV2 vector to deliver *RPE65* gene specific cDNA to the RPE through subretinal injection in patients.^60^ While AAV gene therapy strategies have proven beneficial for patients,^43^^,^^61^^,^^62^^,^^63^^,^^64^^,^^65^ there are three main limitations associated with AAVs: limited DNA packaging capacity (<5 kb), manufacturing challenges, and concerns regarding immune responses, including inflammatory responses (redness, swelling, pain), cellular immune responses, and theoretical integration-related immune responses.^65^ These limitations underscore the pressing need to develop next-generation gene delivery vehicles for ocular gene therapy.

LNPs are promising next-generation gene delivery vehicles for ocular gene therapy.^66^^,^^67^^,^^68^ Their sizes fall within the nanometer range from approximately 10 nm–200 nm, facilitating easy internalization into cells through cell endocytosis.^68^ LNP offers several safety advantages over viral vectors: (1) LNPs are typically less immunogenic, and it has less possibilities trigger immune responses in the body, (2) LNPs do not integrate into the host DNA and do not have the potential risk of insertional mutagenesis, (3) LNPs can be designed to target specific tissues or cells, minimizing off-target effects and reducing the risk of systemic toxicity, and (4) the simpler manufacturing process of LNPs decreases the risk of contamination and variability in production, enhancing their safety profile.^67^^,^^68^ LNPs can be easily implemented by changes in molecular structure, and they have the ability to carry large molecular DNA or mRNA with up to 20 kb molecular weight,^14^ which makes them well-suited for the delivery of the ocular genes (e.g., *ABCA4* or *USH2A*) that exceed the packaging capacity of virus vectors. LNPs are one of the most powerful synthetic delivery systems for nucleic acid delivery, which could eliminate the drawbacks of viral vectors.

### LNPs are one of the most advanced nonviral vectors

**Figure 3:**
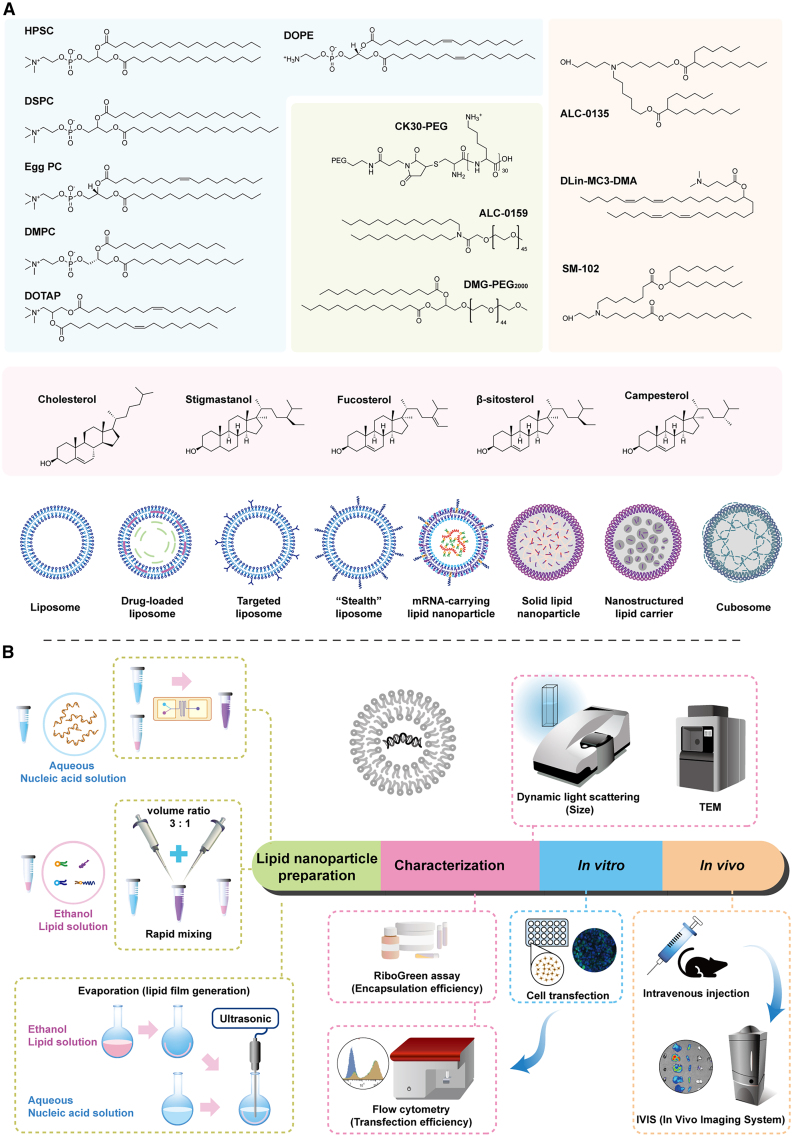
LNP components, assembly and characterization methods Schematic representation of different types of lipid nanocarriers and the common lipids (A) used to assemble them, as well as the manufacturing and characteristic process of LNPs (B).

LNPs are one of the most advanced nonviral clinically approved small-molecule and nucleic acid delivery systems.^69^^,^^70^ However, the concept or definition is not uniform. In some articles or reviews, scientists classified LNPs as lipid-containing nanocarriers (Figure 3A), including liposomes, solid LNPs (SLNs), nanostructured lipid carriers (NLCs),^71^ or cationic lipid-nucleic acid complexes.^72^^,^^73^ However, some scientists strictly limited the definition of LNPs to cationic (or ionizable) lipid-nucleic acid complexes,^68^ which are typically composed of the following four types of lipids or biomolecules: (a) cholesterol, which is a stabilizing agent, (b) natural phospholipids, which support the lipid bilayer morphology, (c) lipid-conjugated PEG, which enhances the half-life of LNPs, and (d) an ionizable lipid or lipid polymer, which enhances self-assembly into virus-sized particles and induces the endosomal release of mRNA into the cytoplasm.^68^ In this review, we use looser concepts of LNPs to broadly sketch out a more comprehensive perspective of the application of LNPs for ocular gene therapy.

#### Compositions and manufacturing of LNPs

The primary components of LNPs are lipids. These lipids include natural phospholipids, such as phosphatidylcholine, phosphatidylethanolamine, and phosphatidylserine. Additionally, cholesterol or its analogues (β-sitosterol, fucosterol, campesterol, and stigmastanol) is often used as a stabilizing agent to maintain the integrity of the lipid bilayer. To enhance the stability and circulation time of LNPs in the bloodstream, lipids can be conjugated with PEG, a process known as PEGylation. PEGylation helps to reduce the recognition and uptake of LNPs by the immune system, increasing their half-life. LNPs also contain ionizable lipids or lipid polymers, which enable the LNPs to package and protect the therapeutic payload (such as siRNA or mRNA) and facilitate endosomal release once LNPs are inside the target cells. Ionizable lipids ((6Z,9Z,28Z,31Z)-heptatriaconta-6,9,28,31-tetraen-19-yl 4-(dimethylamino) butanoate [DLin-MC3-DMA; MC3], heptadecan-9-yl 8-((2-hydroxyethyl)(6-oxooctyl)amino)octanoate [SM-102], 6-((2-hexyldecanoyl)oxy)-N-(6-((2-hexyldecanoyl)oxy)hexyl)-N-(4-hydroxybutyl)hexan-1-aminium [ALC-0315], etc.) carry a positive charge at an acidic pH, promoting endosomal escape and cytoplasmic delivery of the payload. LNP preparation can be done in many ways, such as extrusion of lipid vesicles, rehydration of the lipid film, nanoprecipitation, and microfluidic mixing.^74^^,^^75^ The methods used to generate LNPs are crucial for developing lipid-based nanoformulations with high efficacy because they significantly influence both the size of the LNPs and their encapsulation efficiency.^68^ The manufacturing process (Figure 3B) for the SLN and NLC types of LNPs typically involves rehydrating the lipid film or formation of a water-in-oil emulsion.^76^ The general preparation for these LNPs, including four lipid types, typically entails rapid mixing of aqueous phase containing nucleic acid and organic phase containing lipids components. This mixing process can be achieved through mechanical mixing by pipette, T-junction apparatus, microfluidic methods, and others.^77^ These methods provide a high-throughput and continuous approach for synthesizing nanoparticles from bench scale to clinical volumes. Further comparisons of different manufacturing methods can be found in relevant review articles.^77^^,^^78^^,^^79^

#### The mechanisms of LNPs encapsulate nucleic acids

**Figure 4:**
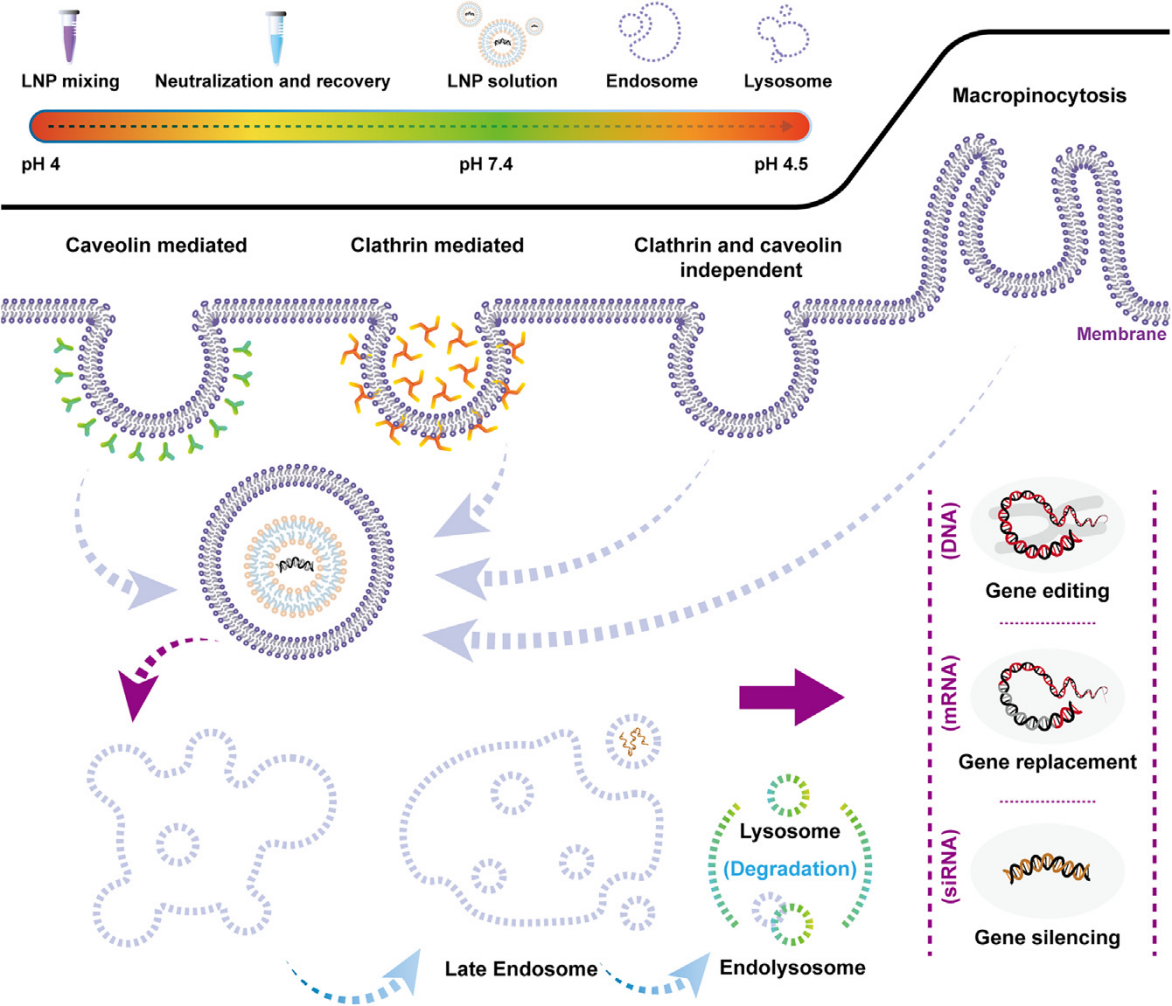
Mechanisms by which LNPs encapsulate nucleic acids and its life cycles

LNPs play a crucial role in safeguarding nucleic acids from degradation and facilitating their entry into targeted cells, thereby exerting therapeutic effects.^76^ The mechanism by which LNPs encapsulate nucleic acids and release them primarily relies on changes in lipid charge and intermolecular interactions at different stages (Figure 4).^75^^,^^76^^,^^77^ First, during the manufacturing process of LNP-nucleic acids, the main components, such as ionizable or cationic lipids, carry a positive charge under acidic conditions. This positive charge is vital for entrapping the negatively charged nucleic acids within the nanoparticles. Second, LNPs maintain an overall neutral surface charge under physiological conditions, achieved through lipids with a specific acid-dissociation constant (pKa). This neutral surface charge protects nucleic acids from degradation by nucleases in physiological fluids. Upon reaching targeted cells, LNPs containing nucleic acids are internalized through various mechanisms, including caveolin-mediated, clathrin-mediated, clathrin and caveolin-independent, and macropinocytic endocytosis. The specific endocytic pathway utilized depends on both the properties of the nanoparticles and the cell type. Once inside the cell, LNPs encapsulating nucleic acids typically become trapped within endosomal compartments, necessitating an endosome escape process before exerting effects in the cytoplasm. This escape is crucial for effective mRNA delivery. While the exact mechanism remains incompletely understood, positively charged lipids may facilitate electrostatic interactions and fusion with negatively charged endosomal membranes, enabling mRNA molecules to leak into the cytoplasm. However, only a fraction of nucleic acids is able to escape. It is reported that optimization of the pKa values of ionizable lipids or adjustment of the type and ratio of PEG lipids (such as 1,2-dimyristoyl-racglycero-3-methoxypolyethylene glycol-2000 [DMG-PEG_2000_] and 1,2-distearoyl-*sn*-glycero-3-phosphoethanolamine-N-[methoxy(polyethylene glycol)-2000] [DMPE-PEG_2000_]) or neutral lipids (such as 1,2-distearoyl-snglycero-3-phosphocholine [DSPC] and 1,2-dioleoyl-*sn*-glycero-3-phosphoethanolamine [DOPE]) can enhance endosomal escape.^80^ Regardless of the material and the mechanism, the efficiency of endosomal escape remains relatively low, which calls for the implementation of novel methods to capture the intricacies of endosomal escape.^81^

#### Methods used to enhance the stability and cellular uptake of LNPs encapsulate nucleic acids

The development process of LNP-nucleic acids was extremely hard, aiming to surmount complex obstacles, such as how to efficiently and safely deliver nucleic acids to desired tissues and cells and how to enhance the performance of nucleic acids with respect to their activity and stability. First, suitable and precise chemical modifications of nucleic acids according to the natural structure of nucleotides, can increase its inherent efficacy, specificity, and stability.^81^^,^^82^ For instance, (1) chemical modifications by adding methyl groups or backbone modifications by adding phosphorothioate linkages to the DNA^83^^,^^84^; (2) the engineering of mRNA molecules of 5′ cap, a 3′ poly(A) tail, a protein-coding sequence and 5′ and 3′ untranslated regions (UTRs), as well as the design of circular RNA and self-amplified mRNA^80^^,^^85^^,^^86^; and (3) the precise modification of siRNA at the phosphate backbone, the ribose moiety, or the base.^87^^,^^88^ In this review, we mainly focus on the methods for encapsulation of nucleic acids within specific protective carriers, namely, LNPs, to enhance the stability and cellular uptake of nucleic acids.

The components of LNPs have a significant influence on the stability and cellular uptake efficiency of LNP-nucleic acids. Typically, the ionizable lipid in an LNP plays a major role in protecting nucleic acids and facilitating their cytosolic transport,^89^ optimization of the chemical structure, and identities of ionizable lipids in LNPs that could enhance the fluidity and delivery efficiency of LNP-nucleic acids.^80^^,^^89^ For example, the ionizable lipid, 1,2-dilinoleyloxy-N, N-dimethyl-3-aminopropane was originally synthesized for siRNA delivery,^90^ and its delivery efficacy was enhanced by modifying the linker and hydrophobic regions, resulting in 2,2-dilinoleyl-4-dimethylaminoethyl-[1,3]-dioxolane (DLin-KC2-DMA).^91^ Further optimization of the amine head group of DLin-KC2-DMA led to MC3,^92^ which is a key component for delivering patisiran.^17^ LNPs containing MC3 have been investigated for mRNA therapeutics. Mitchell et al.^89^ have categorized ionizable lipids into five primary types and provided a detailed description of how these ionizable lipids can impact the transfection efficiency of LNP-nucleic acids.

The primary effects of the structural lipids (phospholipid and cholesterol) and the PEG-lipid in LNPs is to provide particle stability, control over size, and blood compatibility. Adjustments of the quantities in the formulation and chemical properties of these lipids could also enhance the efficiency of LNP-nucleic acid. For instance, Oberli et al.^93^ found that LNPs containing DSPC, a phosphatidylcholine with saturated tails, performed better than those containing DOPE, a phosphoethanolamine with two unsaturated tails, in C57BL/6 mice by subcutaneous injection. Sahay Gaurav’s group has done a lot of works related to LNP containing PEG-variants and cholesterol analogs in the recent years,^94^^,^^95^^,^^96^^,^^97^ One of their studies showed that introducing an ethyl group at the C-24 position of cholesterol significantly enhanced mRNA transfection efficacy in HeLa cells. This improvement was observed through a comparison of transfection efficacy among various LNPs containing 22 different cholesterol analogs with diverse head, body, and tail structures, all tested in HeLa cells.^98^

In addition to optimizing the components of LNPs to improve the stability and cellular uptake efficiency of LNP-nucleic acids, adjusting the storage format is also a viable option to enhance the long-term stability of LNP-nucleic acids. Several investigators demonstrated that LNP-mRNA vaccines could be lyophilized.^86^^,^^99^^,^^100^^,^^101^ Muramatsu et al.^101^ reported that lyophilized firefly luciferase-encoding mRNA-LNPs could maintain their high expression, and no decrease in the immunogenicity of a lyophilized mRNA-LNP vaccine was observed after 12 weeks of storage at room temperature or for at least 24 weeks after storage at 4°C in comparative mouse studies. Higuchi et al.^102^ summarized the research that utilized a lyophilization method to enhance the shelf life and stability of LNP-nucleic acids. Achievement of high stability and cellular uptake of LNP-nucleic acids still needs further exploration, as the cellular uptake efficiency could be influence by cell type, and how to improve the specific to targeted cells is also a hot topic.

This review offers an overview of lipid-containing nanoparticles (SLNs, NLCs, and nanoparticles containing cationic or ionizable lipids) as innovative ophthalmic drug delivery systems designed to enhance the bioavailability of drugs in ocular tissues for the treatment of eye diseases.

### Preclinical development of lipid nanotechnology-mediated gene therapy

#### DNA delivery of lipid nanotechnology for long-term gene therapy

**Figure 5:**
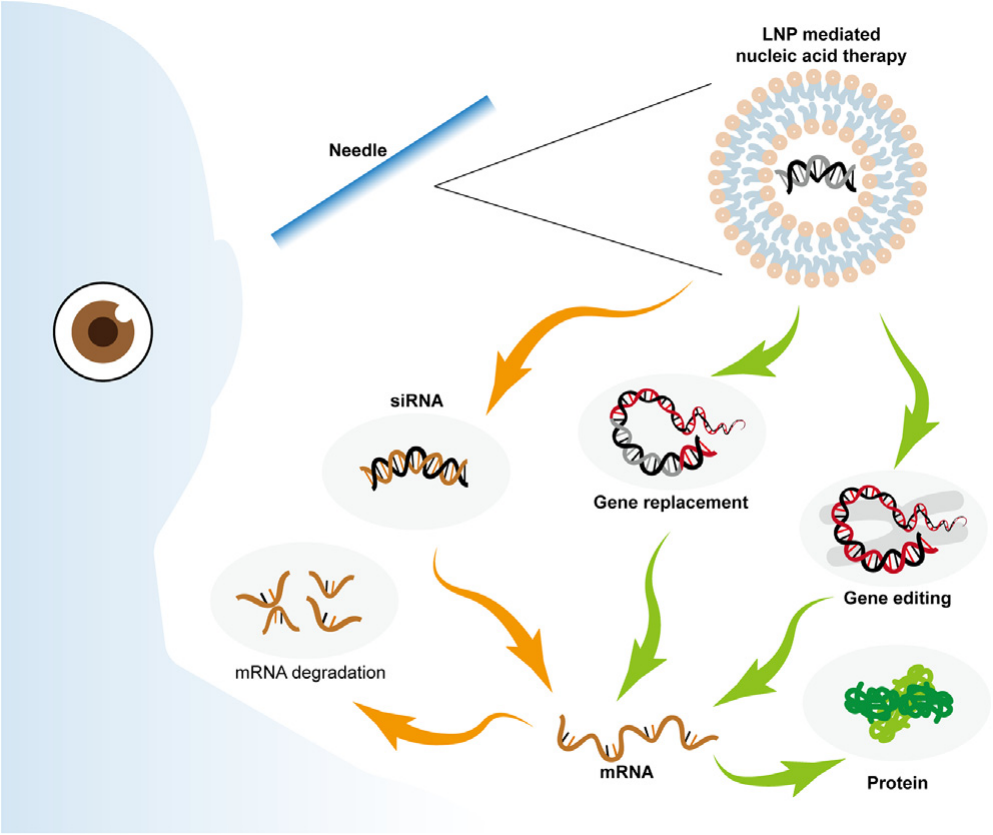
Schematic representation of gene therapies including gene replacement, gene silencing, and gene editing in the application of ocular disease

**Figure 6:**
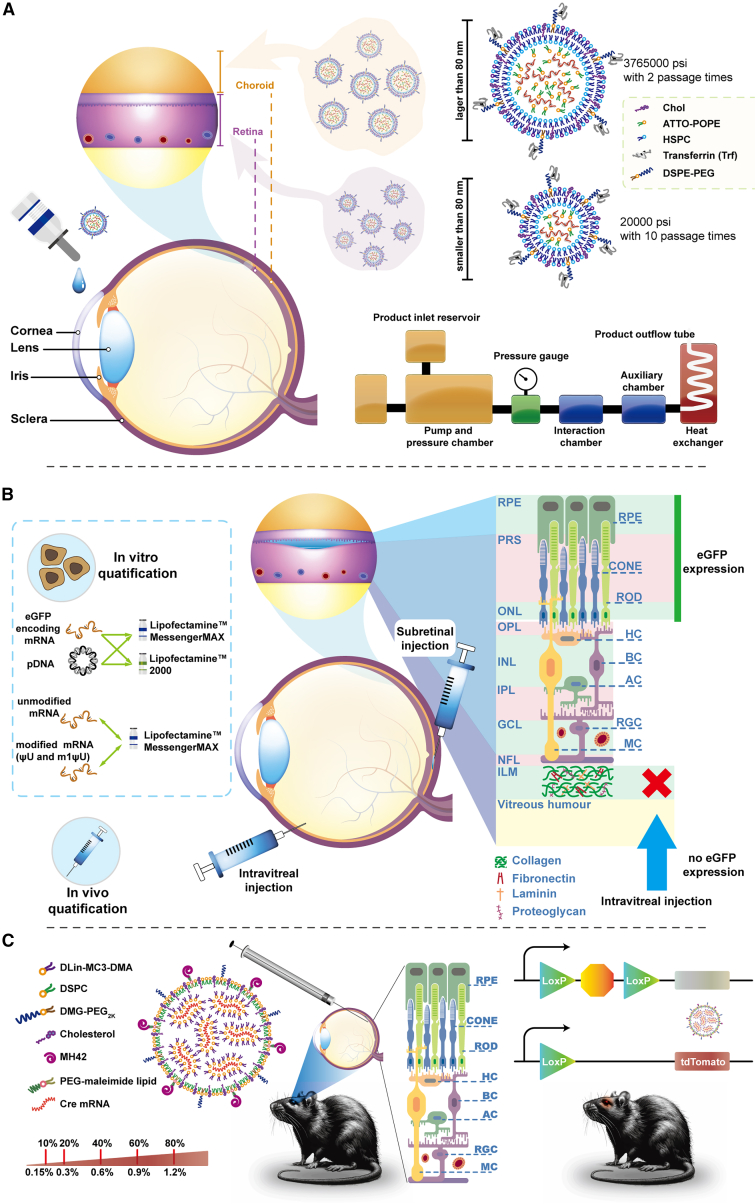
Representative research on LNP-based gene therapeutics in ocular diseases (A) Schematic diagram of DSPE-PEG-Trf micelles based on pDNA therapies, which was drawn according to the outline of experiments reported by Lajunen et al.^104^ (B) Schematic diagram for mRNA-loaded lipid-based carrier-induced gene therapies, which were drawn according to the outline of experiments reported by Devoldere et al.^115^ (C) Schematic of peptides conjugated LNP-mRNA formulation.

Nanoparticle-based gene delivery in the eyes has primarily centered around DNA as the preferred therapeutic agent (Figure 5).^15^^,^^103^^,^^104^^,^^105^^,^^106^^,^^107^^,^^108^^,^^109^^,^^110^^,^^111^^,^^112^^,^^113^^,^^114^ Overcoming the challenge of delivering drugs by topically applied eye drops to the posterior segment of the eyes remains a significant hurdle in the field of ocular drug development. Lajunen et al.^104^ developed methods (Figure 6A) for liposome preparation utilizing a microfluidizer under high pressure, which allows for the attainment of adjustable nanoparticle sizes, even less than 80 nm, and demonstrates a high loading capacity of plasmid DNA (pDNA) within the liposomes. HSPC (L-α-phosphatidylcholine)/cholesterol/DSPE-PEG (1,2-distearoyl-sn-glycero-3-phosphoethanolamine-N-[amino(PEG)-2000])/fluorescent ATTO-DOPE (1,2-dioleoyl-sn-glycero-3-phosphoethanolamine-Atto 647N) in molar ratios of 2:1:0.02:0.005 were dissolved in chloroform as free lipids to prepare liposomes, and luciferase pDNA was entrapped in the lipids by the double emulsion method manufactured by the microfluidizer. Transferrin (Trf) was selected as the targeting ligand to the RPE and was further included in liposomes to form DSPE-PEG-Trf micelles. The distribution of DSPE-PEG-Trf micelles to different posterior segment tissues was observed to be size dependent following topical eye drop application.^104^ Smaller DSPE-PEG-Trf liposomes (with a diameter of 68 ± 5 nm) produced a distinct fluorescence signal in the RPE layer, whereas larger liposomes (with a diameter of 100 ± 13 nm) exhibited a weaker signal in the choroid layer. This study demonstrates the potential of active targeting W/O/W double emulsion as eye drops for delivering drugs or pDNA to posterior segment tissues of the eye, specifically targeting the RPE and choroid regions.^104^ Farjo et al.^14^^,^^105^ assessed the transfected efficiency of compacted DNA nanoparticles for ocular tissues, and a rod-shaped compacted DNA nanoparticles was generated by combining pDNA with synthesized CK_30_PEG10K that was composed of a 30-mer cationic polylysine and 10-kDa PEG through a maleimide linkage.^14^^,^^105^ The CK30PEG10K derived DNA nanoparticles exhibited a small minor diameter, ranging from 8 to 11 nm, which contributed to transfer the compacted DNA into the nuclei of retinal cells. Han et al.^15^^,^^16^ reported that CK30PEG-derived DNA nanoparticles resulted in marked correction of functional and structural improvements in *Abca4*^−/−^ mouse models related to Stargardt disease. This correction was manifested by improved recovery of dark adaptation and a reduction in lipofuscin granules.^15^^,^^16^

SLNs are characterized by a solid lipid matrix enveloped by a layer of surfactants within an aqueous dispersion.^116^ These nanoparticles are widely acknowledged as one type of the most powerful lipid-contained vectors.^117^ Delgado et al.^110^ measured the expression of enhanced green fluorescent protein (eGFP) in the ocular tissues of rats treated with a nonviral vector, dextran-protamine-DNA-SLN complex, bearing the reporter gene pCMS-eGFP. In later works, Apaolaza et al.^112^ designed two types of nonviral vectors based on SLNs, the dextran–protamine-SLN complex and the hyaluronic acid-protamine-SLN complex, which were used as carriers of the pDNA (pCAG-*GFP*_CMV-*RS1*) that encodes the GFP and retinoschisin proteins. The findings demonstrated successful transfer of the *RS1* gene to *Rs1h*-deficient animals using nonviral vectors based on SLNs, marking the first instance of this successful gene transfer,^112^ which demonstrated nonviral gene therapy as a feasible future therapeutic tool for retinal disorders. In this study, gene expression was observed in multiple cell types of the retina (RPE, PRs), even those that do not endogenously express retinoschisin in wild-type animals, such as GCs. Thus, Apaolaza et al.^113^ constructed a new plasmid that contained the *RS1* gene driven by the specific murine opsin promoter-targeted heterologous gene expression to PR in follow-up research, and dextran-protamine-SLN and hyaluronic acid-protamine-SLN complexes were used. Hyaluronic acid-protamine-SLN resulted in a significantly higher increase in the thickness of both the retina and outer nuclear layer.^113^

In addition to targeting XLRS, a gemini surfactant-based lipidic (GL)-NP was developed and complexed with pDNA (pCMV-tdTomato) for the treatment of glaucoma.^114^ The plasmid-encapsulated GL-NP complex (P-GLNP) obtained a polymorphic structure, which was biocompatible with RGCs and significantly enhanced the transfection efficiency of pDNA *in vivo*.^114^ P-GLNPs were found to accumulate within the nerve fiber layer of the retina after intravitreal injection in C57BL/6N mice. The study concluded that GLNPs can be used successfully for the targeted delivery of pDNA to glaucoma tissues such as the ciliary body, trabecular meshwork, and damaged retina.^114^

The delivery of pDNA by LNPs in preclinical studies is still limited by the lack of systematic structural and functional studies on DNA-loaded LNPs (Table 2).^118^ Quagliarini et al.^118^ prepared 16 multicomponent DNA-loaded LNPs by microfluidics. These LNPs were formulated with different lipid composition, surface functionalization, and manufacturing factors.^118^ After screening various DNA-loaded LNPs formulations, LNP15 emerged as the optimal candidate for further validation due to its exceptional balance between high transfection efficiency and favorable biocompatibility. LNP15 was composed of a mixture of four lipid compositions (DOTAP:DC-Chol:CHOL:DOPE) at percentages of 13.3:40:13.3:32. The formulation was prepared with a total flow rate of 2 mL/min, resulting in a diameter of 129 ± 3 nm. Notably, its superior performance is attributed to a disordered nanostructure characterized by small, unoriented layers of pDNA nestled between closely apposed lipid membranes, which undergo significant destabilization upon interaction with cellular lipids.^118^ Their results provide new insights into the structure-activity relationship of pDNA-loaded LNPs and pave the way to the clinical translation of this gene delivery technology.^118^

**Table 2:** Representative preclinical researches related to LNP carrying DNA drugs encoding eye diseases related genes

LNP types and compositions	Genetic materials	Transfected cells *in vitro*	Targeted ocular diseases	*In vivo* administration	Animal model	*In vivo* distribution	Reference
PEG-substituted polylysine	*ABCA4* cDNA	–	Stargardt dystrophy	subretinally deliver	*Abca4*-deficient mice	rod and cone outer segments	Han et al.^15^
DSPE-PEG-Trf micelles	luciferase pDNA	–	–	eye drops	male SD-rats	diameter (68 ± 5 nm) permeated to the RPE; diameter (100 ± 13 nm) distributed to the choroidal endothelium.	Lajunen et al.^104^
PEG-substituted lysine peptides	pZEEGFP5.1	–	–	intravitreal; subretinal	Balb/cJ mice	transfected nearly all of the PR cells	Farjo et al.^105^
SLN includes Precirol® ATO 5, DOTAP, Tween 80	pCMS-EGFP	ARPE-19; HEK293	retinal gene therapy	–	–	–	Del Pozo-Rodríguez et al.,^106^ del Pozo-Rodríguez et al.,^107^ Delgado et al.^108^
SLN includes protamine/dextran/hyaluronic acid and polyvinyl alcohol	pcDNA3-EGFP, pUNO1-hIL10 plasmid	human corneal epithelial cells	corneal inflammation	topical administration	wild-type and *IL-10* knockout mice	mainly transfected corneal epithelial cells	Vicente-Pascual et al.^109^
Dextran–protamine–DNA–SLN complex	pCMS-EGFP DNA, pCEP4-RS1 DNA	ARPE-19	XLRS	intravitreal, subretinal, and topical administration	Wistar rats	topical application: transfect corneal cells	Delgado et al.^110^
SLN includes Precirol® ATO 5, DOTAP, Tween 80	pCMS-EGFP DNA, pCEP4-RS1 DNA	ARPE-19, HEK293, PR cell	XLRS	intravitreal administration	retinoschisin *Rs1h*-deficient mice	retinal and outer nuclear layer	Apaolaza et al.^111^
Gemini surfactant-phospho LNPs (GL-NPs)	Cy5 labeled pDNA	rat RGCs	non-invasive glaucoma	intravitreal or topical application	C57BL/6N mice	intravitreal injection--the nerve fiber layer of retina, topical application: several anterior chamber tissues	Alqawlaq et al.^114^
DX-SLN or HA-SLN	pCAG-GFP_CMVRS1 DNA	ARPE-19	XLRS	intravitreal or subretinal. injection	*Rs1h*-deficient mice	retinal layer	Apaolaza et al.^112^
DX-SLN and HA-SLN	pCMVGFP_mOPS-RS1 DNA	661W	XLRS	intravitreal administration	*Rs1h*-deficient mice	retinal and outer nuclear layer	Apaolaza et al.^113^

mOPS, murine opsin promoter.

The lipid-DNA complex can undergo endosomal internalization,^58^ initiating endosomal membrane destabilization, which causes a switch of the anionic lipids present on the cytoplasmic side and the subsequent formation of charge-neutralized ion pairs.^119^ Lipid-based vectors may use clathrin-mediated endocytosis and micropinocytosis pathways,^120^ their mechanism depending on lipid dose and charge ratio. The size and charge of lipids influence cellular uptake and DNA transfer through nuclear pores.^121^ Subretinal injections represent the predominant method for delivering lipid-based DNA nanoparticles in retinal gene delivery. However, the efficiency of transfection with these nanoparticles for the neural retina or RPE is restricted by the nuclear entry necessity in postmitotic retinal cells. Additionally, concerns about toxicity and the safety implications of gene integration need to be addressed.^122^

#### mRNA for gene expression or protein supplement therapy

mRNA typically comprises five essential elements: a 5′ cap, a 3′ poly(A) tail, a protein-coding sequence, and 5′ and 3′ UTRs. These components are pivotal for mRNA function and degradation regulation.^80^ Traditional mRNA is prone to degradation by ribonucleases (RNases) in the cytoplasm, which limits their half-life and effectiveness as therapeutic agents. The advancement of *in vitro* transcribed mRNA addresses this issue by offering avenues for mRNA engineering through strategies including nucleotide modifications and cap analogs.^115^^,^^123^^,^^124^ For example, substituting natural ribonucleotides with modified ones like pseudouridine (Ψ)^85^ or 5-methylcytidine, and modifying the 5′ cap structure with 7-methylguanosine cap analogues or analogues with extended linker molecules contributes to elevated gene expression levels. Additionally, these modifications mitigate immune responses and enhance mRNA stability, thereby prolonging its half-life.^125^ This progress in mRNA technology is paving the way for therapeutic protein production at levels that are increasingly considered for gene editing using endo/exonucleases or activating transcription factors for regenerative applications.^126^ This approach is particularly advantageous in scenarios where the transient presence of the protein is desired. It is highly desirable to develop synthesis vectors that can help to transfer mRNA-mediated therapeutics, as well as protect mRNA from degeneration by RNase enzymes and prolong circulation time. Several research groups have studied the potential of the application of LNP-delivered mRNA in the ocular field.^72^^,^^97^^,^^115^^,^^123^^,^^127^^,^^128^

RP comprises a group of hereditary and degenerative ocular diseases resulting from specific gene mutations in the rod and/or cone PRs and RPE.^66^ Currently, no effective pharmacological treatments are available for patients with RP; however, a few clinical trials involving supplements of vitamin A and E have been conducted, some cases demonstrated a slower progression of the RP disease.^72^ In this field, Devoldere et al.^115^ explored the potential of chemically stabilized mRNA using the commercially available transfection agents Lipofectamine 2000 and Lipofectamine MessengerMAX for ocular applications for the first time. More specifically (Figure 6B), they conducted an investigation into the transfected effects of lipid-based carriers delivered mRNA on MIOM1 Müller cells and ARPE-19 cells (*in vitro*). Meanwhile, C57BL6/J mice and conventional bovine retinal explants were used for *in vivo* and *ex vivo* experiments individually. The results showed that the expression of eGFP in MIOM1 Müller cells and ARPE-19 cells treated with lipid-carried mRNA was significantly stronger than that with lipid-carried pDNA. Moreover, lipid-carried m1ψU-modified mRNA exhibited approximately 1,800-fold higher eGFP expression compared with lipid-carried pDNA.^115^ Notably, eGFP expression could be detected for at least 20 days after a single administration of m1ψU-modified mRNA *in vitro*. Additionally, they identified that the inner limiting membrane is one of the significant barrier for nonviral delivery of mRNA, which trapped mRNA complexes on the vitreal side.^115^ This study demonstrated the potential of mRNA-mediated therapy for retinal diseases for the first time.^115^

In the same year (2019), Patel et al.^127^ prepared an ionizable lipid (MC3) containing LNPs to entrap eGFP or mCherry coding mRNA. The LNP-mRNA were applied through subretinal injections to albino BALB/c mice with posterior eye diseases (RP), and kinetics and localization of PR expression were studied. Their findings revealed that LNP-based mRNA primarily transfected the RPE and exhibited limited distribution in the Müller glia after subretinal injections.^127^ The kinetics of gene expression following treatment with LNP-mRNA were rapidly detectable within 4 h and sustained for 96 h in albino BALB/c mice.^127^

Ryals et al.^97^ conducted a study on cell-specific LNP-mRNA that enables various dosing regimens and exhibits low immunogenicity. These characteristics are desirable for broadening the applicability of LNP-mRNA in retinal applications. Eight LNP-mRNAs encoding luciferase were prepared by changing the PEG-lipids (DMG-PEG) from 5% to 0.5%; their size ranged from 50 nm to 150 nm, and MC3 was used as an ionizable lipid in each LNP-mRNA. They found LNP-mRNA containing 0.5% PEG elicited the highest expression after subretinal injections, and its size measured around 150 nm. In addition, they also demonstrated that LNPs that delivered Cre-mRNA or luciferase mRNA induced cell-specific protein expression in Ai9 mice after different administration routes.^97^ More specifically, LNP-mRNA was mainly transfected into the RPE after subretinal injection. However, after intravitreal injection, LNP-mRNA was mainly transfected into the Muller glia, the optic nerve head, and the trabecular meshwork, no luciferase expression could be observed in RPE. Additionally, they explored the mechanisms of LNP-mRNA encoding mCherry by subretinal injection into the eyes of *ApoE*^−/−^ and *Mertk*^−/−^ mice.^97^ The results of RPE transfection indicated that the intracellular delivery of LNP-mRNA is not solely dependent on apolipoprotein adsorption or phagocytosis.^97^ However, the detailed delivery mechanism is unclear and needs further investigation.

Most studies have demonstrated that LNP-mRNA delivery is primarily limited to the RPE and Müller glia.^97^^,^^115^^,^^127^^,^^128^ LNPs face the challenge of overcoming ocular barriers after subretinal or intravitreal injection to transfect neuronal cells and PRs, which are critical for visual phototransduction.^115^ To achieve this goal, Herrera-Barrera et al.^123^ developed peptide-conjugated LNPs that can enable mRNA delivery to the neural retina (Figure 6C), expanding the utility of LNP-mRNA therapies for inherited blindness. In particular, they innovatively employed a combinatorial M13 bacteriophage-based heptameric peptide phage display library to explore peptide ligands targeting PRs, the most promising peptide candidates were identified. DLin-MC3-DMA (ionizable lipids), DSPC, cholesterol, and DMG-PEG_2000_ were selected and mixed at molar ratios of 50:38.5:10:1.5. These components were mixed with screened peptide candidates at a molar ratio of 10:1 (peptide:PEG) to generate the peptide-conjugated LNPs. Both the *in vitro* and *in vivo* results demonstrated that the top-performing peptide ligand (MH42; SPALHFLGGGSC)-decorated LNPs could deliver mRNA to the targeted PRs in the eyes of mice and nonhuman primates.^123^

**Figure 7:**
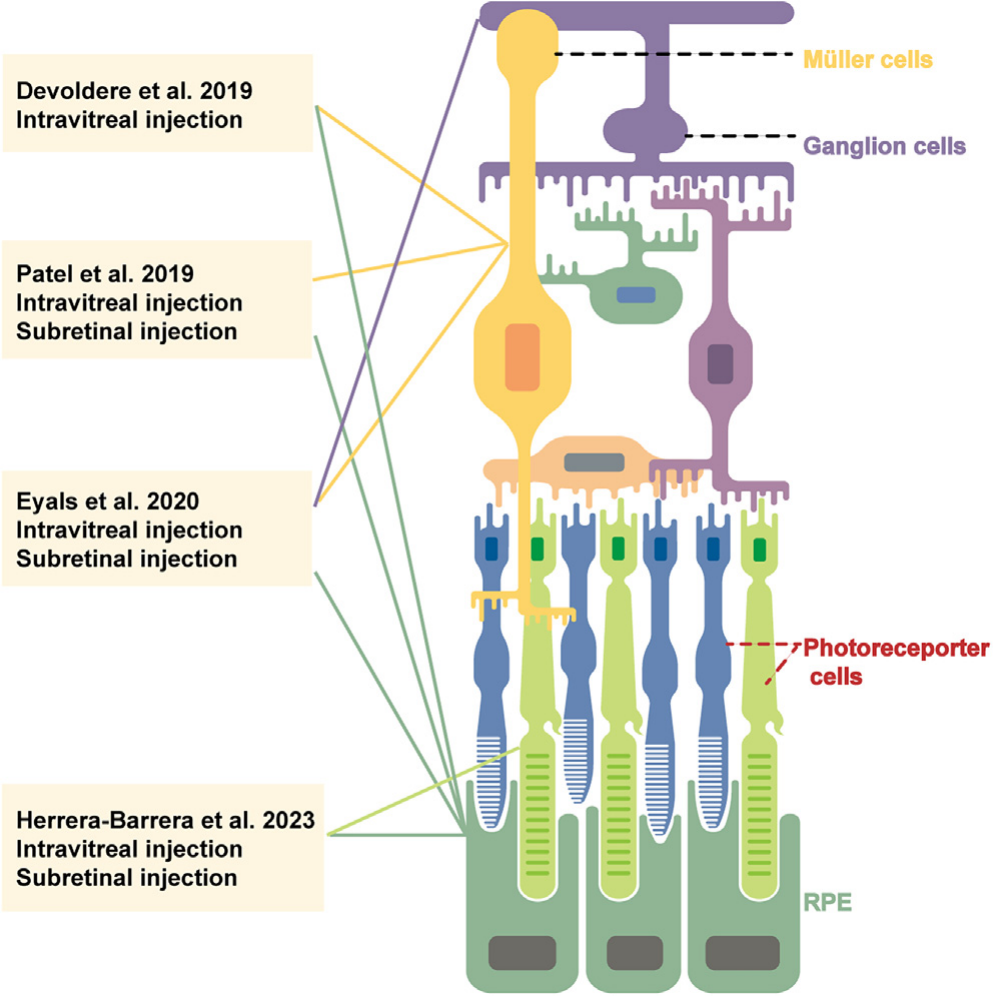
Representative research on LNP-based mRNA in ocular diseases and the distribution of delivered mRNA after intravitreal or subretinal injection

These studies focused on the kinetics and localization of LNP-mRNA therapies applied in ocular diseases (Figure 7). These studies unequivocally demonstrate the identification of mutated genes associated with various ocular diseases, and the use of nonviral LNP-based mRNA therapy has gained significant momentum in the field of ocular gene therapy.

#### siRNA delivery by LNPs for gene silencing

SiRNA molecules are negatively charged, making it challenging for them to readily cross tightly packed and hydrophobic cell membranes. Consequently, carriers are employed to facilitate their delivery. These carriers are designed to safeguard therapeutic siRNA from degradation, filtration, and phagocytosis in the bloodstream.^129^ Additionally, carriers facilitate transport across the vascular endothelial barrier and diffusion through the extracellular matrix, promote cellular uptake, and allow cargo release into the cytosol.^130^ Here, we enumerate some LNPs that deliver therapeutic siRNA for several ocular diseases.

a) **Posterior capsule opacification (PCO) treatment:** PCO is the most common complication of extracapsular cataract surgery, which is caused by the proliferation of residual lens epithelial cells (LECs) that can be regulated by the mammalian target of rapamycin (mTOR). Zhang et al.^131^ used Lipofectamine 2000 to deliver siRNA that specifically attenuates mTOR (simTOR) into human lens epithelial B3 cells (HLE B3). SimTOR delivered by Lipofectamine 2000 was found to effectively inhibit mTOR/p70S6K/Akt in LEC proliferation, and it also suppressed mTOR complex 1 (mTORC1), mTORC2, and transforming growth factor β-induced epithelial-to-mesenchymal transition.^131^
b) **AMD:** Vascular endothelial growth factor A (VEGF-A) is widely recognized as a pathological initiator in certain conditions, such as AMD. A phase 2 study was undertaken to assess the effectiveness of various dosing regimens of PF-04523655 (PF), an siRNA drug, with or without ranibizumab (a VEGF-A antagonist), in individuals with neovascular AMD.^132^ PF functions by decreasing VEGF-A production through the suppression of the expression of the hypoxia-inducible gene *RTP801*, consequently inhibiting the activation of the mTOR pathway. The findings indicated that when combined with ranibizumab, PF led to an average improvement in best-corrected visual acuity that exceeded that achieved with ranibizumab monotherapy.^132^ Small synthetic novel RNA oligonucleotides (nkRNA and PnkRNA) targeting mouse VEGF were transfected into a mouse endothelial cell line (UVfemale2) by commercial HiPerFect (a unique mixture of cations and neutral lipids; Qiagen, Valencia, CA), and they inhibited AMD-associated angiogenesis via the Toll-like receptor 3-independent pathway.^133^ Additionally, siRNA targeting poly(ADP-ribose) polymerase-1 (PARP-1) was transfected into ARPE-19 cells using Lipofectamine RNAiMAX reagent. This approach contributed to the effectiveness in regulating retinal cell apoptosis in a dry AMD animal model system, resembling the effect of the pharmacological inhibitor of PARP-1, olaparib.^134^
c) **Choroidal neovascularization:** PEGylated liposome-protamine-hyaluronic acid nanoparticles (PEG-LPH-NP-S) loaded with siRNA were investigated for the treatment of choroidal neovascularization in ARPE19 cells and a laser-induced rat disease model.^135^ PEG-LPH-NP-S loaded with siRNA effectively inhibited the expression of vascular endothelial growth factor receptor-1 and decreased the choroidal neovascularization area compared with naked siRNA or PEG-LPH-NP with negative siRNA. The toxicity of PEG-LPH-NP-S on the rat retina was found to be not significant.^135^
d) **Corneal neovascularization (CNV):** Strategies for suppressing the expression of metalloproteinase 9 (*MMP-9*) in corneal cells^136^ or delivery of siVEGF into human umbilical vein endothelial cells to knock down VEGF expression *in vitro* and *in vivo* indicated positive effects on the treatment of CNV and its associated inflammation. Torrecilla et al.^136^ Designed an SLN system to deliver short hairpin RNA (*p*-shRNA-MMP-9; 4993 bp) that downregulate *MMP-9.* The SLN-based shRNA were able to downregulate the *MMP-9* expression in HCE-2 cells thus inhibiting cell migration and tube formation.^136^ Liu et al.^137^ prepared a novel reactive oxygen species (ROS)-responsive LNP (ROS-TK-5) for siRNA delivery into corneal lesions for enhanced RNAi as a potential CNV treatment. The siRNA nanocomplexes effectively knocked down VEGF expression and suppressed CNV formation in an alkali burn model after subconjunctival injection.^137^ These results showcase the potential of LNPs as effective gene delivery systems for treating inflammation associated with of CNV-associated inflammation through RNAi technology.^136^^,^^137^
e) **Fibrosis prevention after glaucoma filtration surgery:** The use of lipid-peptide nanoparticles encapsulating siRNA targeting the myocardin-related transcription factor (MRTF)-B has shown promising outcomes.^138^ This approach effectively silenced the *MRTF-B* gene, leading to a reduction in scar tissue formation in the conjunctiva. Fernando et al.^138^ screened 15 lipid-peptide-siRNA (LPR) nanoparticles formulated with different lipid compositions, surface charges, and targeting or nontargeting peptides. The LPR formulation of the DOTMA/DOPE lipid with the targeting peptide Y (K_16_GACYGLPHKFCG), which is designed as LYR nanoparticles, demonstrated the highest efficiency in silencing the *MRTF-B* gene compared with the nontargeting formulation in human conjunctival fibroblasts. The developed LYR nanoparticles successfully decreased conjunctival scarring in a rabbit glaucoma model, and no local or systemic adverse side effects were reported.^138^

Several other investigators have explored the RGD-labeled liposomes carrying *VEGF*-siRNA for gene therapy to treat retinopathy. The RGD-labeled liposomes efficiently delivered siRNA to RPE cells via integrin-mediated endocytosis, providing a viable option for targeting genes to RPE cells.^139^^,^^140^^,^^141^ Overall, significant progress has been made to drive siRNA into different eye diseases treatment. However, the delivery of therapeutic siRNA to induce the potent and specific silencing of genetic targets in target cells remains one of the greatest challenges in RNAi therapy. Furthermore, the gene-silencing effect of siRNAs is transient, typically effective only for 3–7 days, as siRNAs are degraded by tissue nucleases.^139^^,^^140^^,^^141^

## Conclusions and future perspectives

The field of LNP-based nucleic acid delivery is a rapidly evolving research field with significant potential for the development of new and improved therapies for various ocular diseases. However, there is a noticeable gap in research focused on LNP-mRNA delivery compared with LNP-DNA or LNP-siRNA delivery. Additionally, visual functional evaluation studies specifically for LNP-mRNA therapies are currently lacking. This research gap presents a valuable opportunity for further exploration into LNP-mediated delivery of mRNA encoding genes associated with eye diseases. For instance, investigating LNP-(*RPE65*) mRNA for LCA2, LNP-(*MERTK*) mRNA for *MERTK* mutation-associated RP, or LNP-(*PDE6B*) mRNA for inherited retinal dystrophies could open new avenues for therapeutic development. Preclinical trials assessing the safety and efficacy of LNP-based mRNA therapies for these conditions have the potential to offer transformative treatments for patients with limited therapeutic options.

LNPs have demonstrated efficient delivery to RPE cells in numerous studies by far, research focusing on targeted delivery to other retinal cell types, such as PR cells or optic nerve cells, is limited. The lack of cell specificity poses a significant limitation to the efficacy of LNPs. To overcome this challenge, researchers have explored various strategies, including the incorporation of targeting fragments, such as antibodies or ligands into LNP structures to enable targeted delivery. Additionally, recent studies have demonstrated that the modification of PEG-variant surface properties can influence the cellular tropism of LNPs.^94^ For example, LNPs containing negatively charged-carboxyl or carboxy-ester modified PEG-lipids exhibited 27% and 16% PR transfection, respectively, with pan-retinal distribution observed in the PRs and RPE. Conversely, LNPs containing positively charged amine-modified PEG lipids and conventional LNPs showed tdTomato signal exclusively in the RPE following subretinal injections.^94^ This study indicated that the design of new lipids or optimization of lipids with different properties for LNP utilization is also a direction in the future. Moving forward, the development of new lipids or the optimization of existing lipids with distinct properties for LNP utilization represents a promising direction for enhancing the cell specificity and efficacy of LNP-based therapies. By tailoring lipid formulations to target specific cell types or tissues, researchers can unlock the full therapeutic potential of LNPs and advance precision medicine approaches for the treatment of ocular diseases and beyond.

Currently, most ocular gene therapies in preclinical and clinical research rely on intravitreal and subretinal injections, which carry a high risk of inducing inflammation. For mRNA and siRNA treatments, single dosing is often insufficient, necessitating multiple administrations that may exacerbate inflammatory responses. Hence, the development of highly efficient and minimally immunogenic LNPs is a key focus for future research. Exploring the synergistic benefits of combining LNP-based nucleic acid therapies with other modalities, such as anti-VEGF agents, steroids, or gene editing technologies, holds promise for enhancing treatment efficacy and addressing the multifaceted nature of complex eye diseases.

From an industrial standpoint, sustained investment in translational research and clinical trials focused on LNP-based therapies for various eye conditions will be crucial for establishing robust clinical evidence, validating therapeutic effectiveness, and guiding clinical practice. Collaborative efforts, including multicenter trials, real-world evidence studies, and patient registries, can offer valuable insights into the long-term safety and efficacy of these treatments.

Overall, advancing LNP-based nucleic acid therapies in ophthalmology demands interdisciplinary collaboration, innovative research methodologies, and strategic investments to meet unmet clinical needs and enhance patient outcomes. By prioritizing targeted delivery, combination approaches, safety optimization, personalized medicine, regulatory compliance, and translational efforts, the field can leverage the full potential of LNPs to revolutionize the management of ocular disorders.
